# Effects of acute pain and strain of the periodontium due to orthodontic separation on the occlusal tactile acuity of healthy individuals

**DOI:** 10.1007/s00784-021-03971-z

**Published:** 2021-05-05

**Authors:** Rosaria Bucci, Michail Koutris, Vittorio Simeon, Frank Lobbezoo, Ambrosina Michelotti

**Affiliations:** 1 Department of Neurosciences, Reproductive Sciences and Oral Sciences, School of Orthodontics and Temporomandibular Disorder, University of Naples Federico II, Via Pansini 5, 80131 Naples, Italy; 2 Department of Oral Kinesiology, Academic Centre for Dentistry Amsterdam (ACTA), MOVE Research Institute Amsterdam, University of Amsterdam and VU University Amsterdam, Amsterdam, The Netherlands; 3 Department of Public, Clinical and Preventive Medicine, Medical Statistics Unit, University of Campania ‘Luigi Vanvitelli’, Naples, Italy

**Keywords:** Periodontal pain, Periodontal strain, Orthodontic separators, Periodontal sensation, Tactile acuity

## Abstract

**Objectives:**

The aim of this study was to assess whether pain and strain of the periodontal ligament (PDL), induced by orthodontic separation, alter the somatosensory ability to perceive small thicknesses between occluding teeth (occlusal tactile acuity, OTA).

**Methods:**

The OTA was tested at baseline (T0), using 9 aluminum foils (range 8–72 μm), randomly placed between the molar teeth, and 1 sham test (without foil), asking the participants whether they felt the foil between their teeth. Afterwards, orthodontic separators were placed, and subjects were randomly assigned to one of the two experimental groups: Group Pain (GP: 18 males; 14 females mean age 25.22 ± 2.28 years) had separators removed after 24 h; Group Strain (GS: 14 males; 17 females, mean age 24.03 ± 3.06 years) had separators removed after 7 days. The OTA measurement was repeated in both groups immediately after orthodontic separators removal (T1). A within-group comparison (T1 vs T0) was performed for each testing thickness (ANOVA for repeated measurements, with Bonferroni correction for multiple testing) (*p* < 0.005).

**Results:**

GP showed statistically significant reduction of the OTA at T1, as compared to T0, for the thicknesses 24 μm (*p* = 0.004) and 32 μm (*p* = 0.001). No significant reduction was observed in GS (all *p* > 0.005).

**Conclusions:**

Acute periodontal pain tends to disturb the tactile ability of the teeth, while strain of the PDL in absence of painful sensation determines a return to OTA baseline values.

**Clinical relevance:**

The reduction of OTA might explain the uncomfortable occlusal sensation referred by patients during acute periodontal pain.

## Introduction

Tactile acuity is the ability to measure small differences at skin level in tactile spatial thresholds. This ability is typically measured as the two-point discrimination threshold and defined as the minimum distance between two mechanical stimuli simultaneously applied to the skin that can be perceived as two separate points [1, 2]. Translating this concept to the somatosensory functions of the stomatognathic system, the occlusal tactile acuity (OTA) has been defined as the ability to detect small thickness changes between antagonist teeth during maximal intercuspation [3]. This accurate sensory information is crucial for several oral motor behaviors and contributes to refine jaw functions related to biting and chewing. In particular, it plays a primary role in the control of occlusal forces and mandibular movements during the opening reflex of the mandible [4]. This ability mainly relies on the extremely sensitive mechanoreceptors located in the periodontium that are able to detect also very small objects between antagonist teeth [5]. In addition, the numerous nerve endings embedded in the periodontal ligament (PDL) continuously provide sensory feedback about the direction and magnitude of forces applied to each tooth [6, 7]. Therefore, whenever the PDL is absent or damaged, the oral sensorimotor functions might be altered or lost [8].

The PDL is a tissue with high perfusion rate that sustains the surrounding structures. When a prolonged external mechanical force delivered by an orthodontic appliance is applied to a tooth, the compression of the PDL and the activation of its mechanical nociceptors are responsible for an immediate painful response. As a result, inflammatory algogens are released in the PDL causing a delayed and prolonged painful experience [9]. When the mechanical stimulation is protracted in time, the pro-inflammatory chemical mediators in the gingival crevicular fluid rule the bone resorption and reposition process, allowing orthodontic movements of the teeth [10].

The placement of orthodontic separators is considered an unpleasant and painful orthodontic procedure [11]. Separators are usually placed to create space in order to comfortably place an orthodontic band by loosening the tight interproximal contact points between adjacent posterior teeth [12]. It has been reported that the pain and discomfort experienced by patients during tooth separation can vary widely, and patients should be warned that separation placement may adversely affect daily activities like chewing, learning, working, and sleeping [13]. The pain is often described by the patients as feelings of pressure, tension, and soreness of the teeth. It starts immediately after the placement of the separator, presents a peak after 24–36 h, and shows a progressive reduction with complete relief after 5 to 7 days [14, 15]. After 1 week of orthodontic separation, it has been estimated that approximately 0.25 mm of space can be gained between two adjacent teeth, which is the average width of the stretched PDL [16, 17].

One previous study demonstrated that the application of a light mechanical force, by means of specially designed calibrated torque wrench, reduces the proprioceptive and discriminating abilities of the patients for up to 4 days [18]. This might be explained by the fact that the release of interleukin 1β and TNF-α in the PDL induces inflammatory hyperalgesia, characterized by altered neuronal membrane excitability, which can lead to somatosensory alterations [19–21]. Furthermore, studies have shown that also mechanical deformation of a tissue might influence the discriminative tactile acuity, by altering the involvement of sensory receptors [22]. Therefore, due to the primary role of the PDL in the OTA, it can be hypothesized that the acute periodontal pain and mechanical deformation of the PDL might interfere with the OTA.

Therefore, the aim of this study was to evaluate the effects of both periodontal pain and mechanical deformation of the PDL, experimentally obtained with the application of orthodontic separators, on the OTA of adult healthy subjects with natural dentition.

## Materials and methods

This randomized trial was performed according to the Helsinki Declaration and was approved by the local ethical committee of the University of Naples Federico II (Protocol no. 337/18).

### Participants

Volunteers were recruited from among university students and staff of the University of Naples Federico II. Inclusion criteria were as follows: age more than 18 years, willingness to participate in the study, full permanent dentition (excluding third molars), and presence of a visible contact in the molar area. The following conditions were considered exclusion criteria: absence of first permanent molars, removable denture wearers, presence of severe malocclusion (such as severe II class or severe III class, crossbite), ongoing orthodontic treatment, spaces in the upper dentition, presence of large restorations, endodontic treatment, fixed dental prosthesis or implants on the first permanent molars, and/or use of drugs active on the nervous system, such as antiepileptics or anti-Parkinson.

### OTA measurement

A protocol used in previous studies was applied [3, 23, 24]. Briefly, at the baseline (T0) participants were tested for the ability to discriminate 10 different interdental thicknesses: 9 aluminum foils ranging from 8 to 72 μm and one sham test without any foil. The testing thicknesses were placed at the area of the first permanent molars, preferably in correspondence of the mesio-labial cusp, and they were presented 10 times in random order (100 total tests). The participants were asked to close their mouth gently and to indicate if they felt the aluminum foil between their teeth or not. Each answer (YES/NO) was recorded on a spreadsheet. To avoid any additional information, the participants were asked to keep their eyes closed, the cheek mucosa was distanced with a mouth mirror, and headphones with white noise were used to hide the sound of the foils. The participants were naive to the existence of a sham test.

### Orthodontic separator placement

**Fig. 1:**
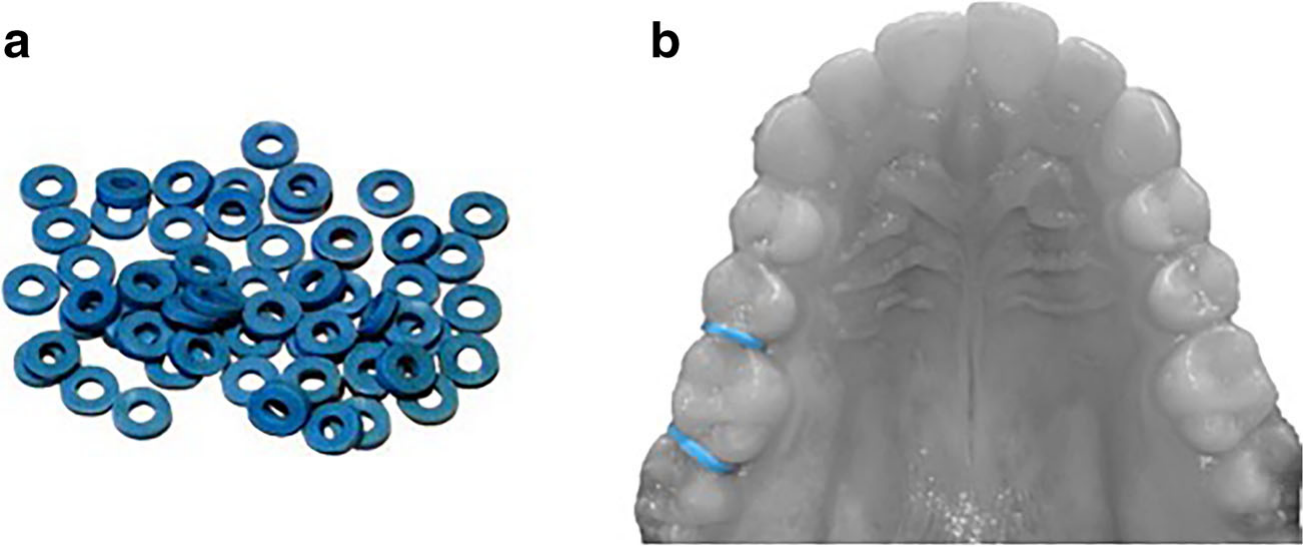
Orthodontic separators outside of the mouth (**a**) and placed between tooth 1.6 and neighbor teeth (**b**)

**Fig. 2:**
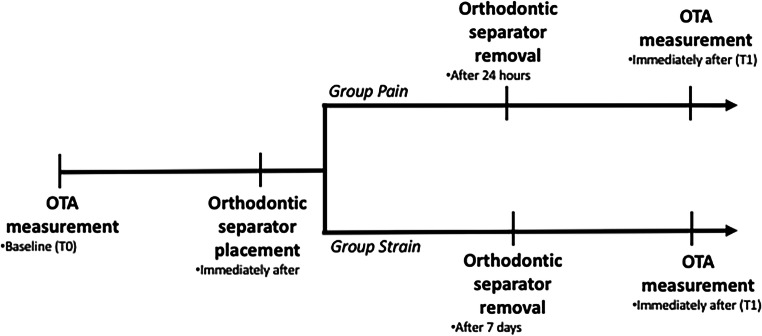
Timeline of the experimental procedures in the two experimental groups. Group Pain (GP) had the orthodontic separators removed after 24 h, when the peak of periodontal pain was expected. Group Strain (GS) had the orthodontic separators removed after 7 days, when elastic deformation of the periodontal ligament in absence of nociceptive stimuli was expected

Immediately after the first OTA measurement, two separator elastics (American Orthodontics, Sheboygan, WI, USA) were placed between the upper first molar and the adjacent teeth. Conventionally, the separators were placed on the right side (Fig. 1), unless the first upper molar right presented some exclusion criteria. Afterwards, the patients were randomly assigned, using black envelopes, to one of the two following groups. The first group had the orthodontic separators removed after 24 h (Group Pain—GP). The second group had the orthodontic separators removed after 7 days (Group Strain—GS). Considering the time course of pain related to the use of orthodontic separators [13, 25, 26], a condition of experimentally induced acute periodontal pain was tested in GP, while GS resembled mechanical strain of the PDL fibers after cessation of the painful stimulus. In both groups, the OTA measurement was repeated immediately after the removal of the orthodontic separators (T1). The timing of the experimental procedures, in the two groups, is shown in Fig. 2.

### Statistical analysis

Considering the repetition of the measurements, mean percentage of correct answers (YES when the foil was present and NO when the foil was absent) was calculated for each thickness tested (mean %). Within each group, analysis of variance (ANOVA) for repeated measurement with foil thickness (from 0 to 72 μm) as repeated factor and time (T0 and T1) was computed. Furthermore, difference was evaluated for each foil thickness and, after Bonferroni correction, statistical significance was set at *p* < 0.005 (alfa: 0.05/10 = 0.005). Considering the mean percentage of correct answers as the main outcome, to achieve a power of 80% with a significance level of 5%, a sample of 60 participants was necessary to detect an effect size between the two time points of 0.5 (large effect size) using an F test for repeated measures ANOVA.

## Results

Sixty-three (63) volunteers were recruited in this study: 32 participants were assigned to GP (18 males; 14 females mean age 25.22 ± 2.28 years); 31 participants to GS (14 males; 17 females, mean age 24.03 years ± 3.06).

**Fig. 3:**
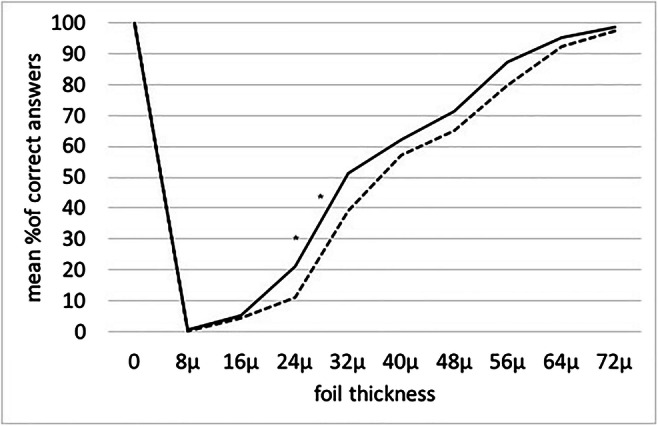
Mean % of correct answers for each thickness tested and for the sham test, in Group Pain (24 h). The response was considered correct if the answer was YES when the foil was present and NO when the foil was absent. * indicates statistically significant difference (*p* < 0.05) in the comparison between time points (T1 vs T1)

In GP, an overall reduction of the OTA was observed immediately after the removal of the orthodontic separator, compared with baseline OTA measurement (F(0,1) = 3.91; *p* = 0.05). In particular, a statistically significant reduction of the OTA (i.e., reduced ability to detect the thickness) was found at T1, as compared to T0, for the thicknesses 24 μm (*p* = 0.004) and 32 μm (*p* = 0.001) (Table 1, Fig. 3).

**Table 1:** Mean % of correct answers and 95% confidence interval (95% C.I.) at baseline (T0) and after the removal of the orthodontic separators (T1) in the Group Pain (24 h). Statistically significant differences between T1 and T0 are reported in bold

Foil thickness	T0 mean%	95% C.I.	T1 mean%	95% C.I.	*p*-value
0 μm	99.7	94.6–100	100	94.0–100	0.932
8 μm	3.7	0–8.8	3.1	0–8.2	0.865
16 μm	7.8	2.7–12.9	4.7	0–9.7	0.395
24 μm	**21.6**	**16.4–26.6**	**10.9**	**5.8**–**16.0**	***0.004***
32 μm	**51.2**	**46.1**–**56.3**	**39.1**	**33.9**–**44.2**	***0.001***
40 μm	62.2	57.1–67.3	57.5	52.4–62.6	0.202
48 μm	70.9	65.8–76.0	65.3	60.2–70.4	0.126
56 μm	87.5	82.4–92.6	80	74.9–85.1	0.041
64 μm	93.3	90.2–100	93.1	88.0–98.2	0.552
72 μm	98.4	93.3–100	97.5	92.4–100	0.798

**Fig. 4:**
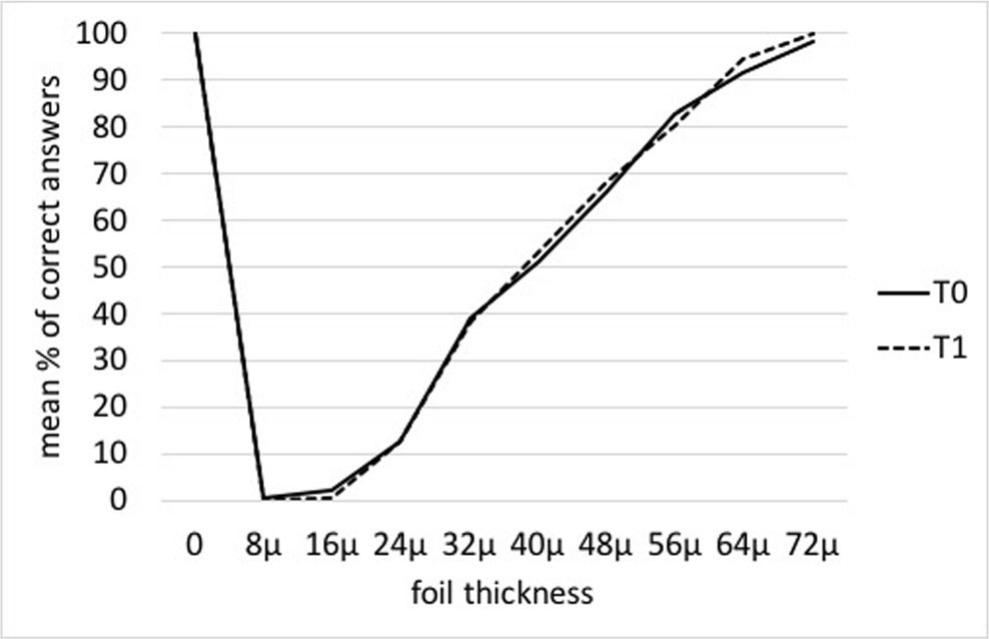
Mean % of correct answers for each thickness tested and for the sham test, in Group Strain (7 days). The response was considered correct if the answer was YES when the foil was present and NO when the foil was absent

In GS, the OTA trend was unchanged at T1 as compared to T0 (F(0,1) = 0.98; *p* = 0.77), and no statistically significant results were observed in the T1 vs T0 comparison (all *p* > 0.005; Table 2, Fig. 4).

**Table 2:** Mean % of correct answers and 95% confidence interval (95% C.I.) at the baseline (T0) and after the removal of the orthodontic separators (T1) in the Group Strain (7 days)

Foil thickness	T0 mean%	95% C.I.	T1 mean%	95% C.I.	*p*-value
0 μm	100	95.5–100	100	95.5–100	1.000
8 μm	0.32	0–4.5	0.60	0–4.5	1.000
16 μm	2.25	0–6.7	0.64	0–5.1	0.617
24 μm	12.9	8.4–17.4	13.9	9.4–18.3	0.764
32 μm	38.7	34.2–43.2	39.03	34.5–43.5	0.920
40 μm	52.9	48.4–57.4	55.8	51.3–60.3	0.368
48 μm	65.8	61.3–70.3	67.4	62.9–71.9	0.617
56 μm	82.2	77.8–86.7	79.3	74.9–83.3	0.368
64 μm	92.2	87.8–96.7	95.8	91.3–100	0.271
72 μm	99.3	94.9–100	100	95.5–100	0.798

## Discussion

The objective of this study was to evaluate the changes in the somatosensory ability to detect small thickness between occluding teeth (named occlusal tactile acuity—OTA) due to acute periodontal pain induced by the placement of orthodontic elastic separators in a group of healthy volunteers. The separators were adopted as experimental model to elicit acute periodontal pain and elastic elongation of the PDL, with complete reversibility. In particular, in one group (Group Pain—GP) separators were removed 24 h after placement, in order to resemble acute periodontal pain, while in the other group (Group Strain—GS) the separators were removed 7 days after placement, in order to obtain mechanical deformation of the PDL along with cessation of the painful sensation. In agreement with the hypothesis, the results showed that the acute periodontal pain yielded a reduction of the somatosensory ability, as pointed out by the significant reduction of OTA at T1, as compared to T0, in GP. On the other hand, no significant findings were observed in GS, supporting that only mechanical strain of the PDL, without nociceptive stimuli, does not affect the OTA.

Orthodontic separators are used in everyday orthodontic practice in order to create interproximal separation, generally between molars and premolars. The obtained space allows adequate placement of molar bands that anchor a fixed orthodontic appliance. The placement of different types of orthodontic separators (brass wire, elastomeric, spring type steel separators, and latex elastics) results in a painful experience for almost all patients [12, 25]. The intensity of pain, as for any kind of pain, presents large inter-individual variability depending on anatomical, psychological, and biological factors. In particular, anxiety and catastrophizing are listed among the factors that can strongly influence pain perception, explaining why some individuals are more sensitive than others to the same painful stimulus [25]. Despite the different intensity, the time course of pain related to the use of separators seems to be highly repeatable, and it has been extensively described in the literature [13, 25, 26]. Therefore, orthodontic separators have been widely used to apply experimental, temporary, and reversible periodontal pain in volunteers with the aim to study the mechanisms underlying the pain using repeatable and standardized stimuli [27].

The application of a mechanical force on a tooth, such as the force exerted by the orthodontic separator placement, determines substantial alterations in the PDL. Periodontal nerve endings consist of low-threshold mechanoreceptors, mainly Ruffini-like, and high-threshold nociceptors that are silent but not inactive under physiological conditions [28]. Histological studies have shown that, when sustained pressure is applied to a tooth, mechanoreceptors present reduced conduction velocity [29, 30], while the recruitment of nociceptive nerve fibers increases due to chemical modulation [31]. The increase in concentration of inflammatory neuropeptides and the augmented manifestation of nociceptors are responsible for the painful response associated with orthodontic tooth movement [28]. Therefore, the complex mechanism of inflammation and healing that occurs in the PDL when a mechanical force is applied, and the interplay between mechanical and nociceptive nerve fibers, might act as a confounding factor for the detection of thicknesses. This mechanism might explain the reduction of the OTA observed in the current study during acute periodontal pain. Similar findings have been observed in a study authored by Soltis and co-workers [18], pointing out that the proprioceptive discrimination of the force intensity applied on incisor was altered by the insertion of orthodontic forces. Recently, Sampaio and co-workers [32] assessed the effects of orthodontic separators on the extra-oral somatosensory functions by means of a standardized battery of quantitative sensory testing. The authors found no changes at extra-oral trigeminal innervation, supporting that somatosensory alterations following periodontal pain are local phenomena and that regions remote from the teeth are not significantly affected. Interestingly, significant differences of tactile perception in the pain group have been observed for 24 μm and 32 μm foils. Previous studies have shown that, in natural dentition, the minimum interdental detection threshold ranges between 10 and 30 μm [33–37]. Hence, whenever an interference in the OTA is present, it is likely to be observed mainly in the smallest thicknesses range.

Recently, the OTA was measured with the same method in a group of individuals affected by facial pain due to temporomandibular disorders (TMD) [3]. Interestingly, the results pointed out an increase of OTA in TMD individuals compared to TMD-free subjects. These findings underline different effects on the somatosensory system of a mostly nociceptive peripheral pain (periodontal pain) compared to a predominantly central pain (TMD pain) in which neuroplastic changes, such as enlargement of receptive fields at the spinal level, might take action. Studies on nociceptive pain in different body areas (such as low back pain induced by saline injection) [2] reported that tactile acuity, measured as two-point discrimination, was decreased during acute pain. These results, in accordance with the current findings, support that nociceptive pain itself causes deterioration of tactile acuity, without involving significant cortical reorganization. One possible mechanism underling this phenomenon is the “touch-gate,” acting similarly to the gate-control theory of pain [38]. In particular, the activation of the nociceptive system and the stimulation of the brain area associated to the pain response might hamper and delay the transmission of the tactile stimuli, thus lowering the discriminating ability [2].

Interestingly, in both study groups (GP and GS) the ability to detect the absence of the foil was unchanged after the application of the separators. This finding suggests that adequate attention was provided by the participants during the experimental sessions. The primary role of attention in detection tasks has been underlined in previous studies using brain imaging. In fact, it has been proven that conscious perception of a sensory stimulus depends not only upon the stimulus intensity, but also on the state of the brain that modulates the reaction to the stimulus [39, 40].

After the insertion of the orthodontic separators, interproximal contact points between adjacent teeth gradually reduce their initial tightness. It has been estimated that, on average, after 1 week of separation the elastic elongation of the PDL provides approximately 0.25 mm of space between two adjacent teeth. Immediately after the removal, relapse of the teeth to the initial contact points’ tightness begins [17]. The displacement within the periodontal ligament is the first step of the tooth movement, without involvement of the cellular cascade that determines the bone resorption process. Tooth movement occurs when an orthodontic force exceeds the bioelastic limits of the supporting tissue, causing a local inflammation. The PDL does not act as a simple spring, but instead tooth displacement elicits a viscoelastic response of the PDL, with an initial squeezing of extracellular fluid towards the alveolus followed by a second mechanism of tightening of the fiber bundles [41]. Therefore, GS experimentally resembles a condition in which the PDL fibers are mechanically stretched, but the pain stimulus has disappeared. In the skin, it has been pointed out that mechanical deformation of the cutaneous layers might alter the tactile acuity measured as two-point discrimination [22, 42]. In the trigeminal region, it was observed that increased intensity of mechanical stimuli applied with ascending monofilaments sizes yielded augmented tactile acuity [43]. Interestingly, when the PDL was mechanically stretched, the findings of the current study pointed out a return to the initial perception ability, with no significant differences compared to the baseline, further supporting the disturbance caused by the nociception in the periodontal area. Differently from skin studies in which the stimulus itself, applied with different forces or shapes, determines a deformation of the studied area, the mechanical deformation of the PDL, involving the embedded mechanoreceptors, occurs gradually during the 7 days of application of the orthodontic separators. The elastic adaptation of the periodontal fibers to the new enlarged conformation might explain why no significant differences were observed in the OTA after the removal of the separators in GS.

Impaction of foreign bodies (dental floss, toothpick, rubber dam), food impaction, or presence of defective restorations has been listed among the possible cause of periodontal discomfort due to pressure, ischemia, and inflammation of the PDL [44]. It is common clinical experience that patients presenting these conditions also refer uncomfortable dental occlusion. The findings of the current study, with the significant reduction of the OTA 24 h after the application of the orthodontic separators, might partially explain this transitory alteration in the dental sensation.

The study presents some limitations. Firstly, the pain rating was not measured. Therefore, it was impossible to determine whether the OTA impairment was correlated with the amount of pain perceived. Knowing whether the amount of pain was correlated with OTA impairment might have a clinical implication, because increased alteration in the occlusal tactility might be expected in patients experiencing higher pain levels. Secondly, due to ethical considerations, two independent groups were compared and no cross-over design was allowed to avoid repetition of the placement of the orthodontic separators in the same subject. Ideally, to obtain a direct comparison, the same individuals should undergo both experimental settings (24 h and 7 days), with a washout period in between, in order to reduce the possible influence of inter-individual differences in the OTA. Thirdly, when the subjects were invited to close their mouth in order to report whether they felt the aluminum foil between their teeth or not, the bite force during the mouth closing task could not be standardized. Therefore, different pressures applied on the molars due to interindividual difference in the bite force might have interfered with OTA measurements. However, all participants were invited to gently close their mouth with light dental contact, so this is not likely to have affected the difference between the two groups. Finally, as already mentioned, the orthodontic separation is an experimental method to study the behavior of the orthodontic pain nociception and of the PDL deformation, but future study on prolonged orthodontic forces is needed to determine whether pain due to the insertion of a fixed orthodontic appliance and mechanical deformation of the PDL due to orthodontic tooth movement determine any changes in the OTA.

## Conclusion

Periodontal pain, induced by orthodontic separation with elastomeric devices, tends to disturb the tactile ability of the teeth, with a significant decrease of perception of small thicknesses. On the other hand, when the PDL is stretched but the pain and the inflammation of the supporting tissue are healed, the OTA returns to baseline values.

## References

[1] Adamczyk W, Luedtke K, Saulicz E (2018). Lumbar tactile acuity in patients with low back pain and healthy controls: systematic review and meta-analysis. Clin J Pain.

[2] Adamczyk WM, Saulicz O, Saulicz E, Luedtke K (2018). Tactile acuity (dys)function in acute nociceptive low back pain: a double-blind experiment. Pain.

[3] Bucci R, Koutris M, Palla S, Sepúlveda Rebaudo GF, Lobbezoo F, Michelotti A (2020). Occlusal tactile acuity in temporomandibular disorders pain patients: a case-control study. J Oral Rehabil.

[4] Jacobs R, van Steenberghe D (1994). Role of periodontal ligament receptors in the tactile function of teeth: a review. J Periodontal Res.

[5] Ash M, Ramfjord S (1995) Occlusion. W.B. Saunders Company, Philadephia

[6] Beertsen W, McCulloch CA, Sodek J (1997). The periodontal ligament: a unique, multifunctional connective tissue. Periodontol 2000.

[7] Trulsson M, Johansson RS, Olsson KA (1992). Directional sensitivity of human periodontal mechanoreceptive afferents to forces applied to the teeth. J Physiol.

[8] SIIRILAE HS, LAINE P (1963). The tactile sensibility of the parodontium to slight axial loadings of the teeth. Acta Odontol Scand.

[9] Polat O, Karaman AI, Durmus E (2005). Effects of preoperative ibuprofen and naproxen sodium on orthodontic pain. Angle Orthod.

[10] Ullrich N, Schröder A, Jantsch J, Spanier G, Proff P, Kirschneck C (2019). The role of mechanotransduction versus hypoxia during simulated orthodontic compressive strain-an in vitro study of human periodontal ligament fibroblasts. Int J Oral Sci.

[11] Krishnan V (2007). Orthodontic pain: from causes to management—a review. Eur J Orthod.

[12] Bondemark L, Fredriksson K, Ilros S (2004). Separation effect and perception of pain and discomfort from two types of orthodontic separators. World J Orthod.

[13] Nalbantgil D, Cakan DG, Oztoprak MO, Arun T (2009). Perception of pain and discomfort during tooth separation. Aust Orthod J.

[14] Erdinç AME, Dinçer B (2004). Perception of pain during orthodontic treatment with fixed appliances. Eur J Orthod.

[15] Ngan P, Kess B, Wilson S (1989). Perception of discomfort by patients undergoing orthodontic treatment. Am J Orthod Dentofac Orthop.

[16] Brosh T, Machol IHT, Vardimon AD (2002). Deformation/recovery cycle of the periodontal ligament in human teeth with single or dual contact points. Arch Oral Biol.

[17] Davidovitch M, Papanicolaou S, Vardimon AD, Brosh T (2008). Duration of elastomeric separation and effect on interproximal contact point characteristics. Am J Orthod Dentofac Orthop.

[18] Soltis JE, Nakfoor PR, Bowman DC (1971). Changes in ability of patients to differentiate intensity of forces applied to maxillary central incisors during orthodontic treatment. J Dent Res.

[19] Shen H, Shao S, Zhang J, Wang Z, Lv D, Chen W, Svensson P, Wang K (2016). Fixed orthodontic appliances cause pain and disturbance in somatosensory function. Eur J Oral Sci.

[20] Lv D, Zhang J, Gu X (2016). Transient pain following orthodontic fixed appliances induces sensitization of gingival and periodontal tissues. J Oral Facial Pain Headache.

[21] Sood M, Bhatt P, Sessle BJ (2015). Mechanical and thermal hypersensitivities associated with orthodontic tooth movement: a behavioral rat model for orthodontic tooth movement-induced pain. J Oral Facial Pain Headache.

[22] Bell-Krotoski JA, Buford WLJ (1997). The force/time relationship of clinically used sensory testing instruments. J Hand Ther.

[23] Bucci R, Lobbezoo F, Michelotti A, Orfanou C, Koutris M (2017). Delayed-onset muscle soreness does not influence occlusal sensitivity and position sense of the mandible. J Oral Rehabil.

[24] Bucci R, Koutris M, Lobbezoo F, Michelotti A (2019). Occlusal sensitivity in individuals with different frequencies of oral parafunction. J Prosthet Dent.

[25] Beck VJ, Farella M, Chandler NP, Kieser JA, Thomson WM (2014). Factors associated with pain induced by orthodontic separators. J Oral Rehabil.

[26] Cioffi I, Michelotti A, Perrotta S, Chiodini P, Ohrbach R (2016). Effect of somatosensory amplification and trait anxiety on experimentally induced orthodontic pain. Eur J Oral Sci.

[27] Michelotti A, Farella M, Martina R (1999). Sensory and motor changes of the human jaw muscles during induced orthodontic pain. Eur J Orthod.

[28] Vandevska-Radunovic V (1999). Neural modulation of inflammatory reactions in dental tissues incident to orthodontic tooth movement. A review of the literature. Eur J Orthod.

[29] Long A, Loescher AR, Robinson PP (1996). A histological study on the effect of different periods of orthodontic force on the innervation and dimensions of the cat periodontal ligament. Arch Oral Biol.

[30] Loescher AR, Robinson PP (1991). Characteristics of periodontal mechanoreceptors supplying reimplanted canine teeth in cats. Arch Oral Biol.

[31] Vandevska-Radunovic V, Kvinnsland S, Kvinnsland IH (1997). Effect of experimental tooth movement on nerve fibres immunoreactive to calcitonin gene-related peptide, protein gene product 9.5, and blood vessel density and distribution in rats. Eur J Orthod.

[32] Sampaio FA, Sampaio CRA, Cunha CO, Costa YM, Alencar PNB, Bonjardim LR, Garib D, Garlet GP, Eliav E, Conti PCR (2019). The effect of orthodontic separator and short-term fixed orthodontic appliance on inflammatory mediators and somatosensory function. J Oral Rehabil.

[33] Kogawa EM, Calderon PDS, Lauris JRP (2010). Evaluation of minimum interdental threshold ability in dentate female temporomandibular disorder patients. J Oral Rehabil.

[34] Calderon P d S, Kogawa EM, Corpas L d S (2009). The influence of gender and bruxism on human minimum interdental threshold ability. J Appl Oral Sci.

[35] Jacobs R, van Steenberghe D (1991). Comparative evaluation of the oral tactile function by means of teeth or implant-supported prostheses. Clin Oral Implants Res.

[36] Johansson H, Sojka P (1991). Pathophysiological mechanisms involved in genesis and spread of muscular tension in occupational muscle pain and in chronic musculoskeletal pain syndromes: a hypothesis. Med Hypotheses.

[37] Riis D, Giddon DB (1970). Interdental discrimination of small thickness differences. J Prosthet Dent.

[38] Mendell LM (2014). Constructing and deconstructing the gate theory of pain. Pain.

[39] Hernandez A, Nacher V, Luna R (2010). Decoding a perceptual decision process across cortex. Neuron.

[40] Forschack N, Nierhaus T, Muller MM, Villringer A (2017). Alpha-band brain oscillations shape the processing of perceptible as well as imperceptible somatosensory stimuli during selective attention. J Neurosci.

[41] Bien SM (1966). Hydrodynamic damping of tooth movement. J Dent Res.

[42] Levin LS, Regan N, Pearsall G, Nunley JA (1989). Variations in two-point discrimination as a function of terminal probes. Microsurgery.

[43] Jung J-K, Byun J-S, Choi J-K (2019). The effect of applied force on two-point discrimination threshold in the trigeminal region. J Oral Facial Pain Headache.

[44] Miranda-Rius J, Brunet-Llobet L, Lahor-Soler E (2018). The periodontium as a potential cause of orofacial pain: a comprehensive review. Open Dent J.

